# Di­chlorido­(pyridine-κ*N*)[2,3,5,6-tetra­kis­(pyridin-2-yl)pyrazine-κ^3^ *N* ^2^,*N* ^1^,*N* ^6^]nickel(II)

**DOI:** 10.1107/S2414314621000948

**Published:** 2021-02-02

**Authors:** Kwang Ha

**Affiliations:** a Chonnam National University, School of Chemical Engineering, Research Institute of Catalysis, Gwangju, Republic of Korea; Vienna University of Technology, Austria

**Keywords:** crystal structure, nickel(II) complex, pyridine, 2,3,5,6-tetra-2-pyridyl­pyrazine

## Abstract

In the title complex, [NiCl_2_(C_5_H_5_N)(C_24_H_16_N_6_)], the Ni^II^ ion is six-coordinated in a distorted octa­hedral coordination environment defined by three N atoms of the tridentate 2,3,5,6-tetra-2-pyridyl­pyrazine ligand, one N atom of the pyridine ligand and two Cl^−^ anions, with the latter being mutually *trans*. The complex is disposed about a twofold rotation axis along the *a* axis. The complex molecules are connected in the crystal *via* C—H⋯Cl, C—H⋯N and π–π [closest inter-centroid separation = 3.7446 (14) Å between pyridyl rings].

## Abstract 2

The central Ni^II^ ion has an N_4_Cl_2_ octa­hedral coordination sphere defined by three N atoms of the tridentate 2,3,5,6-tetra-2-pyridyl­pyrazine ligand, one N atom of the pyridine ligand and two Cl^−^ anions.

## Graphical abstract

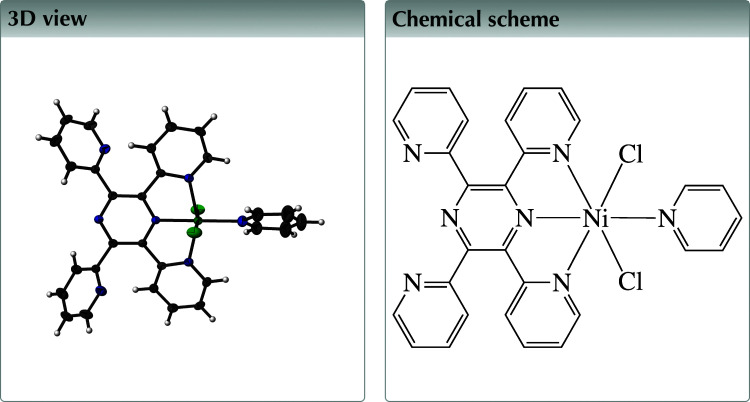

## Structure description

With reference to the title compound, [NiCl_2_(py)(tppz)] (py = pyridine, tppz = 2,3,5,6-tetra-2-pyridyl­pyrazine), the crystal structures of a related tetra­nuclear Ni^II^ complex, [Ni_4_Cl_6_(tppz)_2_(CH_3_OH)_4_]Cl_2_·CH_3_OH (Winpenny *et al.*, 2005 ▸), and of a dinuclear Mn^II^ complex, [Mn_2_Cl_4_(tppz)_2_] (Ha, 2011 ▸), have been determined previously.

**Figure 1:**
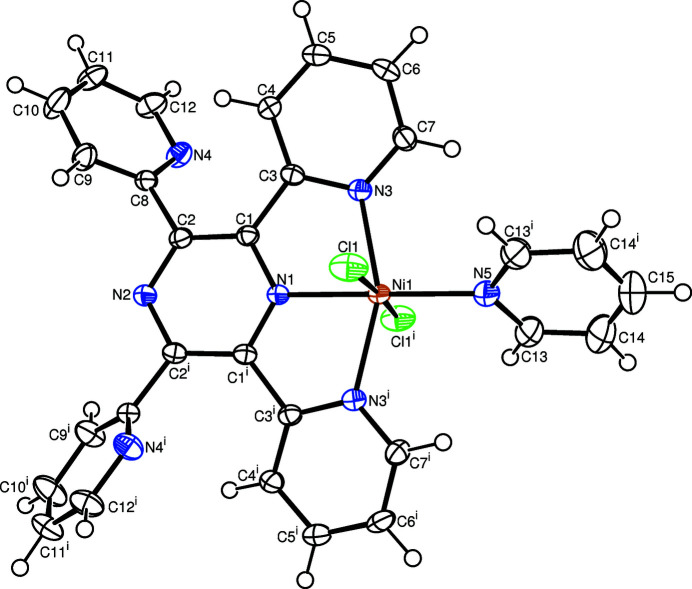
The mol­ecular structure of the title compound showing the atom labelling and displacement ellipsoids drawn at the 50% probability level for all non-H atoms. [Symmetry code: (i) *x* − *y*, −*y*, −*z*.]

In the title complex, the central Ni^II^ cation is six-coordinated in a considerably distorted octa­hedral coordination environment defined by three N atoms of the tridentate tppz ligand, one N atom of the pyridine ligand and two Cl^−^ anions (Fig. 1 ▸). The complex is disposed about a twofold rotation axis along the *a* axis; thus the asymmetric unit contains one half of the complex. The main contribution to the distortion is the tight N—Ni—N chelating angle [<N1—Ni1—N3 = 77.97 (5)°], which results in a non-linear *trans* arrangement of the N3—Ni1—N3^i^ bonds [<N3—Ni1—N3^i^ = 155.95 (11)°; symmetry code: (i) *x* − *y*, −*y*, −*z*], whereas the Cl1—Ni1—Cl1^i^ bonds are almost linear [<Cl1—Ni1—Cl1^i^ = 175.77 (4)°]. The Ni—*N*[pyrazine­(N1) or pyrid­yl(N3, N5)] bond lengths are roughly equivalent, with distances of 2.008 (3) – 2.1026 (19) Å. The pyrazine ring (N1—C1^i^) slightly deviates from planarity, with a maximum deviation of 0.057 (2) Å for the C2 atom from the least-squares plane of the ring. The dihedral angles between the nearly planar pyridyl rings and the least-squares plane of their carrier pyrazine ring are 14.90 (4)° for the coordinating pyridyl ring (N3—C7) and 54.42 (9)° for the non-coord­inating pyridyl ring (N4—C12), respectively. The dihedral angle between the pyrazine ring and the pyridine ligand (N5—C13^i^) is 57.8 (1)°.

**Figure 2:**
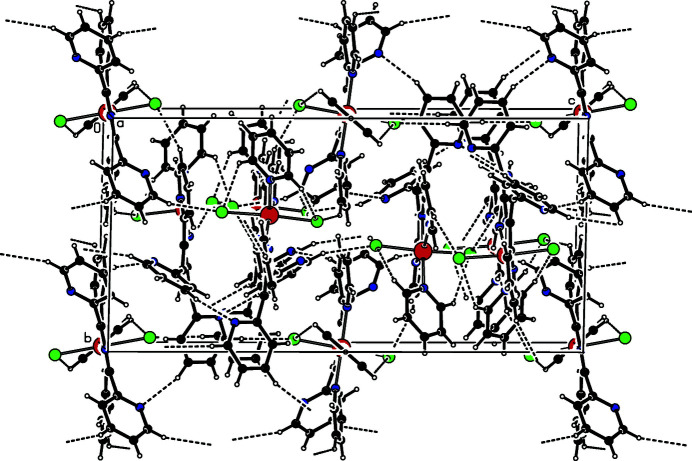
The packing in the crystal of the title compound, viewed approximately along the *a* axis. Hydrogen-bonding inter­actions are drawn as dashed lines. Colour codes are as in Fig. 1 ▸.

In the crystal, the complex displays numerous inter- and intra­molecular π–π inter­actions between adjacent six-membered rings. The most significant inter­action of this kind is that between *Cg*1 (the centroid of the ring N3/C3–C7) and *Cg*1^ii^ [symmetry code: (ii) *x*, *x* − *y*, −*z* + 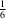 ], with a centroid-to-centroid distance of 3.7446 (14) Å and a dihedral angle between the ring planes of 22.24 (12)°. In addition, the complex exhibits inter- and intra­molecular C—H⋯N and C—H⋯Cl hydrogen bonds (Table 1 ▸) that consolidate the three-dimensional packing (Fig. 2 ▸).

## Synthesis and crystallization

To a solution of NiCl_2_·6H_2_O (0.3779 g, 1.590 mmol) in ethanol (20 ml) was added 2,3,5,6-tetra-2-pyridyl­pyrazine (0.6220 g, 1.601 mmol), followed by stirring for 24 h at rooom temperature. The formed precipitate was separated by filtration, washed with ethanol and acetone, and dried at 323 K, to give a brown powder (0.5045 g). Brown crystals suitable for X-ray analysis were obtained by slow evaporation from its pyridine/*N,N*-di­methyl­formamide (DMF) solution at 333 K.

## Refinement

Crystal data, data collection and structure refinement details are summarized in Table 2 ▸. The maximum and minimum remaining electron density peaks in the difference Fourier map are located 0.34 and 0.74 Å, respectively, from atoms C9 and Ni1.

## Supplementary Material

Crystal structure: contains datablock(s) I. DOI: 10.1107/S2414314621000948/wm4144sup1.cif

Structure factors: contains datablock(s) I. DOI: 10.1107/S2414314621000948/wm4144Isup2.hkl

CCDC reference: 2058988

Additional supporting information:  crystallographic information; 3D view; checkCIF report

## Figures and Tables

**Table 1:** Hydrogen-bond geometry (Å, °)

*D*—H⋯*A*	*D*—H	H⋯*A*	*D*⋯*A*	*D*—H⋯*A*
C6—H6⋯Cl1^i^	0.94	2.78	3.513 (3)	136
C10—H10⋯N4^ii^	0.94	2.46	3.360 (3)	161
C12—H12⋯Cl1^iii^	0.94	2.71	3.602 (3)	160
C13—H13⋯Cl1^iv^	0.94	2.68	3.261 (3)	121

Symmetry codes: (i) 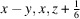 ; (ii) 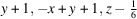 ; (iii) 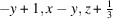 ; (iv) 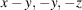 .

**Table 2:** Experimental details

Crystal data	
Chemical formula	[NiCl_2_(C_5_H_5_N)(C_24_H_16_N_6_)]
*M*_r_	597.14
Crystal system, space group	Hexagonal, *P*6_1_22
Temperature (K)	223
*a*, *c* (Å)	13.8244 (4), 23.8935 (8)
*V* (Å^3^)	3954.6 (3)
*Z*	6
Radiation type	Mo *K*α
μ (mm^−1^)	0.97
Crystal size (mm)	0.15 × 0.10 × 0.07

Data collection	
Diffractometer	PHOTON 100 CMOS detector
Absorption correction	Multi-scan (*SADABS*; Krause *et al.*, 2015 ▸)
*T*_min_, *T*_max_	0.700, 0.744
No. of measured, independent and observed [*I* > 2σ(*I*)] reflections	125055, 2614, 2412
*R*_int_	0.106
(sin θ/λ)_max_ (Å^−1^)	0.618

Refinement	
*R*[*F* ^2^ > 2σ(*F* ^2^)], *wR*(*F* ^2^), *S*	0.024, 0.054, 1.08
No. of reflections	2614
No. of parameters	179
H-atom treatment	H-atom parameters constrained
Δρ_max_, Δρ_min_ (e Å^−3^)	0.22, −0.14
Absolute structure	Flack *x* determined using 894 quotients [(*I* ^+^)−(*I* ^−^)]/[(*I* ^+^)+(*I* ^−^)] (Parsons *et al.*, 2013 ▸).
Absolute structure parameter	−0.002 (5)

Computer programs: *APEX2* and *SAINT* (Bruker, 2016 ▸), *SHELXT* (Sheldrick, 2015*a* ▸), *SHELXL* (Sheldrick, 2015*b* ▸) and *ORTEP-3 for Windows* (Farrugia, 2012 ▸).

## full crystallographic data

### Crystal data

**Table:**

[NiCl_2_(C_5_H_5_N)(C_24_H_16_N_6_)]	*D*_x_ = 1.504 Mg m^−^^3^
*M**_r_* = 597.14	Mo *K*α radiation, λ = 0.71073 Å
Hexagonal, *P*6_1_22	Cell parameters from 9199 reflections
*a* = 13.8244 (4) Å	θ = 2.4–26.0°
*c* = 23.8935 (8) Å	µ = 0.97 mm^−^^1^
*V* = 3954.6 (3) Å^3^	*T* = 223 K
*Z* = 6	Block, brown
*F*(000) = 1836	0.15 × 0.10 × 0.07 mm

### Data collection

**Table:**

PHOTON 100 CMOS detector diffractometer	2412 reflections with *I* > 2σ(*I*)
Radiation source: sealed tube	*R*_int_ = 0.106
φ and ω scans	θ_max_ = 26.1°, θ_min_ = 2.4°
Absorption correction: multi-scan (SADABS; Krause *et al.*, 2015)	*h* = −17→17
*T*_min_ = 0.700, *T*_max_ = 0.744	*k* = −17→17
125055 measured reflections	*l* = −29→29
2614 independent reflections	

### Refinement

**Table:**

Refinement on *F*^2^	Secondary atom site location: difference Fourier map
Least-squares matrix: full	Hydrogen site location: inferred from neighbouring sites
*R*[*F*^2^ > 2σ(*F*^2^)] = 0.024	H-atom parameters constrained
*wR*(*F*^2^) = 0.054	*w* = 1/[σ^2^(*F*_o_^2^) + (0.0277*P*)^2^ + 0.9047*P*] where *P* = (*F*_o_^2^ + 2*F*_c_^2^)/3
*S* = 1.08	(Δ/σ)_max_ < 0.001
2614 reflections	Δρ_max_ = 0.22 e Å^−^^3^
179 parameters	Δρ_min_ = −0.14 e Å^−^^3^
0 restraints	Absolute structure: Flack *x* determined using 894 quotients [(*I*^+^)-(*I*^-^)]/[(*I*^+^)+(*I*^-^)] (Parsons *et al.*, 2013).
Primary atom site location: structure-invariant direct methods	Absolute structure parameter: −0.002 (5)

### Special details

**Table:**

Geometry. All esds (except the esd in the dihedral angle between two l.s. planes) are estimated using the full covariance matrix. The cell esds are taken into account individually in the estimation of esds in distances, angles and torsion angles; correlations between esds in cell parameters are only used when they are defined by crystal symmetry. An approximate (isotropic) treatment of cell esds is used for estimating esds involving l.s. planes.

### Fractional atomic coordinates and isotropic or equivalent isotropic displacement parameters (Å^2^)

**Table:**

	*x*	*y*	*z*	*U*_iso_*/*U*_eq_	
Ni1	0.41510 (3)	0.0000	0.0000	0.01866 (12)	
Cl1	0.44000 (6)	0.03687 (5)	−0.09968 (3)	0.03369 (17)	
N1	0.56034 (18)	0.0000	0.0000	0.0181 (6)	
N2	0.75658 (19)	0.0000	0.0000	0.0204 (6)	
N3	0.53182 (16)	0.17007 (16)	0.01208 (8)	0.0196 (4)	
N4	0.86393 (17)	0.24353 (17)	0.07265 (9)	0.0265 (5)	
N5	0.2636 (2)	0.0000	0.0000	0.0303 (7)	
C1	0.65538 (19)	0.09724 (18)	0.00453 (10)	0.0177 (5)	
C2	0.75545 (19)	0.09505 (19)	0.00914 (9)	0.0194 (5)	
C3	0.6391 (2)	0.19584 (19)	0.00471 (10)	0.0187 (5)	
C4	0.7236 (2)	0.30472 (19)	−0.00359 (11)	0.0242 (5)	
H4	0.7967	0.3205	−0.0114	0.029*	
C5	0.6992 (2)	0.3896 (2)	−0.00019 (12)	0.0290 (6)	
H5	0.7557	0.4642	−0.0056	0.035*	
C6	0.5912 (2)	0.3645 (2)	0.01124 (10)	0.0279 (6)	
H6	0.5734	0.4213	0.0157	0.033*	
C7	0.5104 (2)	0.2537 (2)	0.01591 (10)	0.0236 (6)	
H7	0.4362	0.2361	0.0221	0.028*	
C8	0.8647 (2)	0.1935 (2)	0.02484 (10)	0.0199 (5)	
C9	0.9581 (2)	0.2297 (2)	−0.00795 (12)	0.0299 (6)	
H9	0.9555	0.1912	−0.0408	0.036*	
C10	1.0561 (2)	0.3239 (2)	0.00830 (12)	0.0385 (7)	
H10	1.1210	0.3514	−0.0137	0.046*	
C11	1.0565 (2)	0.3761 (2)	0.05722 (11)	0.0323 (6)	
H11	1.1217	0.4403	0.0694	0.039*	
C12	0.9594 (2)	0.3327 (2)	0.08824 (11)	0.0309 (6)	
H12	0.9608	0.3679	0.1221	0.037*	
C13	0.1829 (2)	−0.0605 (2)	0.03686 (14)	0.0414 (7)	
H13	0.1959	−0.1024	0.0638	0.050*	
C14	0.0811 (3)	−0.0642 (3)	0.03700 (18)	0.0579 (10)	
H14	0.0250	−0.1105	0.0624	0.070*	
C15	0.0629 (3)	0.0000	0.0000	0.0639 (15)	
H15	−0.0051	0.0000	0.0000	0.077*	

### Atomic displacement parameters (Å^2^)

**Table:**

	*U*^11^	*U*^22^	*U*^33^	*U*^12^	*U*^13^	*U*^23^
Ni1	0.01813 (17)	0.0145 (2)	0.0222 (2)	0.00723 (11)	0.00093 (9)	0.00185 (17)
Cl1	0.0480 (4)	0.0269 (3)	0.0215 (3)	0.0152 (3)	−0.0012 (3)	0.0025 (2)
N1	0.0175 (10)	0.0147 (13)	0.0210 (14)	0.0073 (7)	−0.0002 (6)	−0.0003 (12)
N2	0.0200 (11)	0.0174 (14)	0.0231 (15)	0.0087 (7)	−0.0006 (6)	−0.0012 (13)
N3	0.0212 (10)	0.0172 (10)	0.0196 (10)	0.0091 (9)	0.0005 (8)	0.0004 (8)
N4	0.0234 (11)	0.0264 (11)	0.0231 (11)	0.0074 (9)	0.0023 (9)	−0.0022 (9)
N5	0.0247 (12)	0.0244 (16)	0.0417 (19)	0.0122 (8)	0.0024 (8)	0.0048 (15)
C1	0.0189 (11)	0.0155 (11)	0.0171 (12)	0.0073 (9)	0.0013 (10)	−0.0007 (9)
C2	0.0195 (12)	0.0165 (12)	0.0194 (12)	0.0069 (10)	0.0017 (10)	0.0010 (10)
C3	0.0220 (12)	0.0163 (11)	0.0176 (11)	0.0095 (10)	−0.0010 (10)	−0.0016 (10)
C4	0.0192 (13)	0.0189 (12)	0.0329 (14)	0.0082 (10)	0.0026 (11)	0.0010 (11)
C5	0.0285 (14)	0.0168 (12)	0.0393 (16)	0.0094 (11)	0.0007 (13)	0.0016 (12)
C6	0.0345 (14)	0.0184 (12)	0.0351 (14)	0.0166 (12)	0.0002 (12)	−0.0012 (11)
C7	0.0257 (13)	0.0237 (13)	0.0254 (14)	0.0153 (11)	0.0025 (10)	0.0015 (10)
C8	0.0186 (12)	0.0160 (12)	0.0241 (13)	0.0080 (10)	−0.0010 (10)	−0.0004 (10)
C9	0.0228 (13)	0.0280 (14)	0.0321 (15)	0.0075 (11)	0.0047 (12)	−0.0085 (12)
C10	0.0206 (14)	0.0381 (16)	0.0406 (18)	0.0025 (12)	0.0078 (12)	−0.0054 (14)
C11	0.0227 (14)	0.0252 (15)	0.0332 (16)	0.0003 (12)	−0.0021 (12)	−0.0018 (12)
C12	0.0319 (14)	0.0260 (14)	0.0232 (13)	0.0057 (12)	−0.0020 (12)	−0.0053 (11)
C13	0.0311 (16)	0.0357 (17)	0.0571 (19)	0.0166 (15)	0.0107 (15)	0.0109 (15)
C14	0.0347 (18)	0.057 (2)	0.082 (3)	0.0222 (17)	0.0195 (18)	0.013 (2)
C15	0.0362 (19)	0.067 (4)	0.098 (5)	0.0336 (18)	0.0005 (18)	0.001 (4)

### Geometric parameters (Å, º)

**Table:**

Ni1—N1	2.008 (3)	C4—H4	0.9400
Ni1—N5	2.094 (3)	C5—C6	1.380 (4)
Ni1—N3^i^	2.1026 (19)	C5—H5	0.9400
Ni1—N3	2.1026 (19)	C6—C7	1.377 (4)
Ni1—Cl1^i^	2.4238 (6)	C6—H6	0.9400
Ni1—Cl1	2.4238 (6)	C7—H7	0.9400
N1—C1	1.334 (3)	C8—C9	1.373 (3)
N1—C1^i^	1.334 (3)	C9—C10	1.385 (4)
N2—C2^i^	1.340 (3)	C9—H9	0.9400
N2—C2	1.340 (3)	C10—C11	1.373 (4)
N3—C7	1.332 (3)	C10—H10	0.9400
N3—C3	1.353 (3)	C11—C12	1.380 (4)
N4—C12	1.332 (3)	C11—H11	0.9400
N4—C8	1.338 (3)	C12—H12	0.9400
N5—C13^i^	1.337 (3)	C13—C14	1.382 (4)
N5—C13	1.337 (3)	C13—H13	0.9400
C1—C2	1.403 (3)	C14—C15	1.362 (4)
C1—C3	1.488 (3)	C14—H14	0.9400
C2—C8	1.489 (3)	C15—C14^i^	1.362 (4)
C3—C4	1.382 (3)	C15—H15	0.9400
C4—C5	1.377 (4)		
			
N1—Ni1—N5	180.0	C5—C4—H4	120.5
N1—Ni1—N3^i^	77.97 (5)	C3—C4—H4	120.5
N5—Ni1—N3^i^	102.03 (5)	C4—C5—C6	119.5 (2)
N1—Ni1—N3	77.97 (5)	C4—C5—H5	120.3
N5—Ni1—N3	102.03 (5)	C6—C5—H5	120.3
N3^i^—Ni1—N3	155.95 (11)	C7—C6—C5	118.0 (2)
N1—Ni1—Cl1^i^	87.89 (2)	C7—C6—H6	121.0
N5—Ni1—Cl1^i^	92.11 (2)	C5—C6—H6	121.0
N3^i^—Ni1—Cl1^i^	87.19 (5)	N3—C7—C6	123.4 (2)
N3—Ni1—Cl1^i^	91.94 (5)	N3—C7—H7	118.3
N1—Ni1—Cl1	87.89 (2)	C6—C7—H7	118.3
N5—Ni1—Cl1	92.11 (2)	N4—C8—C9	123.2 (2)
N3^i^—Ni1—Cl1	91.93 (5)	N4—C8—C2	114.9 (2)
N3—Ni1—Cl1	87.18 (5)	C9—C8—C2	121.9 (2)
Cl1^i^—Ni1—Cl1	175.77 (4)	C8—C9—C10	118.8 (2)
C1—N1—C1^i^	122.5 (3)	C8—C9—H9	120.6
C1—N1—Ni1	118.76 (13)	C10—C9—H9	120.6
C1^i^—N1—Ni1	118.76 (13)	C11—C10—C9	118.6 (2)
C2^i^—N2—C2	119.7 (3)	C11—C10—H10	120.7
C7—N3—C3	118.1 (2)	C9—C10—H10	120.7
C7—N3—Ni1	126.90 (16)	C10—C11—C12	118.7 (2)
C3—N3—Ni1	113.83 (15)	C10—C11—H11	120.6
C12—N4—C8	117.2 (2)	C12—C11—H11	120.6
C13^i^—N5—C13	117.1 (4)	N4—C12—C11	123.4 (3)
C13^i^—N5—Ni1	121.44 (18)	N4—C12—H12	118.3
C13—N5—Ni1	121.44 (18)	C11—C12—H12	118.3
N1—C1—C2	118.0 (2)	N5—C13—C14	122.7 (3)
N1—C1—C3	113.6 (2)	N5—C13—H13	118.7
C2—C1—C3	128.4 (2)	C14—C13—H13	118.7
N2—C2—C1	120.2 (2)	C15—C14—C13	119.4 (4)
N2—C2—C8	115.7 (2)	C15—C14—H14	120.3
C1—C2—C8	124.0 (2)	C13—C14—H14	120.3
N3—C3—C4	121.6 (2)	C14^i^—C15—C14	118.6 (5)
N3—C3—C1	114.0 (2)	C14^i^—C15—H15	120.7
C4—C3—C1	124.4 (2)	C14—C15—H15	120.7
C5—C4—C3	119.1 (2)		
			
C1^i^—N1—C1—C2	−5.20 (15)	C4—C5—C6—C7	−3.4 (4)
Ni1—N1—C1—C2	174.80 (15)	C3—N3—C7—C6	1.8 (4)
C1^i^—N1—C1—C3	175.6 (2)	Ni1—N3—C7—C6	−164.88 (19)
Ni1—N1—C1—C3	−4.4 (2)	C5—C6—C7—N3	2.6 (4)
C2^i^—N2—C2—C1	−5.36 (16)	C12—N4—C8—C9	0.2 (4)
C2^i^—N2—C2—C8	173.8 (2)	C12—N4—C8—C2	−179.4 (2)
N1—C1—C2—N2	10.7 (3)	N2—C2—C8—N4	−125.1 (2)
C3—C1—C2—N2	−170.2 (2)	C1—C2—C8—N4	54.0 (3)
N1—C1—C2—C8	−168.31 (19)	N2—C2—C8—C9	55.3 (3)
C3—C1—C2—C8	10.8 (4)	C1—C2—C8—C9	−125.6 (3)
C7—N3—C3—C4	−5.6 (4)	N4—C8—C9—C10	−1.4 (4)
Ni1—N3—C3—C4	162.80 (19)	C2—C8—C9—C10	178.1 (3)
C7—N3—C3—C1	176.4 (2)	C8—C9—C10—C11	1.3 (5)
Ni1—N3—C3—C1	−15.3 (3)	C9—C10—C11—C12	0.1 (5)
N1—C1—C3—N3	13.1 (3)	C8—N4—C12—C11	1.3 (4)
C2—C1—C3—N3	−166.0 (2)	C10—C11—C12—N4	−1.4 (5)
N1—C1—C3—C4	−164.9 (2)	C13^i^—N5—C13—C14	1.5 (3)
C2—C1—C3—C4	16.0 (4)	Ni1—N5—C13—C14	−178.5 (3)
N3—C3—C4—C5	4.8 (4)	N5—C13—C14—C15	−3.1 (5)
C1—C3—C4—C5	−177.3 (2)	C13—C14—C15—C14^i^	1.5 (3)
C3—C4—C5—C6	−0.2 (4)		

Symmetry code: (i) *x*−*y*, −*y*, −*z*.

### Hydrogen-bond geometry (Å, º)

**Table:**

*D*—H···*A*	*D*—H	H···*A*	*D*···*A*	*D*—H···*A*
C6—H6···Cl1^ii^	0.94	2.78	3.513 (3)	136
C10—H10···N4^iii^	0.94	2.46	3.360 (3)	161
C12—H12···Cl1^iv^	0.94	2.71	3.602 (3)	160
C13—H13···Cl1^i^	0.94	2.68	3.261 (3)	121

Symmetry codes: (i) *x*−*y*, −*y*, −*z*; (ii) *x*−*y*, *x*, *z*+1/6; (iii) *y*+1, −*x*+*y*+1, *z*−1/6; (iv) −*y*+1, *x*−*y*, *z*+1/3.
